# Ticks, rickettsial and erlichial infection in small mammals from Atlantic forest remnants in northeastern Brazil

**DOI:** 10.1016/j.ijppaw.2018.10.001

**Published:** 2018-10-09

**Authors:** Marcos G. Lopes, Sebastián Muñoz-Leal, Julia T. Ribeiro de Lima, Gislene Fatima da S. Rocha Fournier, Igor da Cunha L. Acosta, Thiago F. Martins, Diego G. Ramirez, Solange M. Gennari, Marcelo B. Labruna

**Affiliations:** a Department of Preventive Veterinary Medicine and Animal Health, Faculty of Veterinary Medicine, University of São Paulo, Av. Prof. Orlando Marques de Paiva 87, Cidade Universitária, 05508-270, São Paulo, SP, Brazil; b Mestrado em Medicina e Bem estar animal, Universidade Santo Amaro, Av. Prof. Eneas de Siqueira Neto, 340, São Paulo, 04529-300, Brazil

**Keywords:** *Anaplasmataceae*, *Rickettsia*, *Ehrlichia*, Tick-borne diseases, Wildlife

## Abstract

We evaluated infection by *Rickettsia* spp. and *Ehrlichia* spp in small mammals and their ticks from two Atlantic forest conservation areas in the state of Rio Grande do Norte, northeastern Brazil. A total of 39 small mammals were captured during 2012–2013, encompassing 33 marsupials (29 *Didelphis albiventris*, four *Monodelphis domestica*), three Cricetidae rodents (two *Necromys lasiurus*, one *Rattus rattus*), one Caviomorpha rodent (*Thrichomys apereoides*) and two armadillos (*Euphractus sexcinctus*). The ticks *Amblyomma auricularium, Ixodes loricatus,* and *Ornithodoros mimon* were collected from *D. albiventris,* whereas only *A. auricularium* was collected from armadillos. Through immunofluorescence assay with *Rickettsia* spp. antigens, 6/28 (21%) *D. albiventris* and the single *R. rattus* specimen reacted to at least one rickettsial antigen, with highest seroprevalence and endpoint titers to *Rickettsia amblyommatis.* A total of 150 ticks (126 A*. auricularium,* nine *I. loricatus,* 15 *O. mimon*) was tested for rickettsial infection by PCR, which detected only *R. amblyommatis* in most of the *A. auricularium* ticks. Lung and spleen samples were collected from small mammals (two *N. lasiurus,* six *D. albiventris,* three *M. domestica,* one *T. apereoides,* one *R. rattus*) and were tested by PCR for *Anaplasmataceae* agents. The spleen from one *D. albiventris* contained a new ehrlichial agent, here named as *Ehrlichia* sp. strain Natal. Phylogenetic analysis inferred from the *dsb* gene of *Ehrlichia* spp. indicates that this novel agent is potentially a new species. Future studies should monitor the possible role of rickettsial and/or ehrlichial microorganisms as agents of emerging diseases in these degraded areas of Atlantic forest, just as has occurred with other agents in degraded areas of this biome in southeastern Brazil.

## Highlights

• *Amblyomma auricularium, Ixodes loricatus* and *Ornithodoros mimon* ticks were found.
• Infection by *Rickettsia amblyommatis* was detected in *A. auricularium* ticks.
• Wild small mammals presented serological evidence of *R. amblyommatis* infection.
• A potentially novel *Ehrlichia* species infected the opossum *Didelphis albiventris.*

## Graphical abstract

**Figure:**
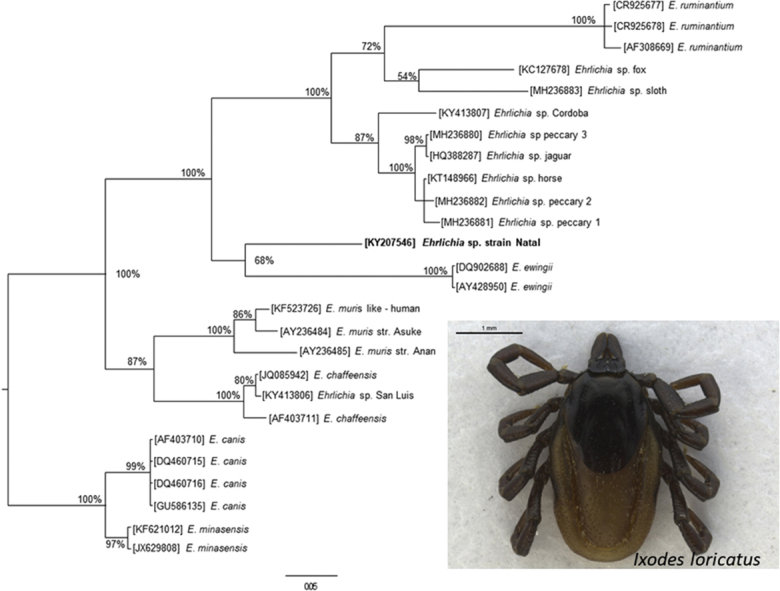

## 1 Introduction

Many bacteria of the genera *Rickettsia* (Rickettsiaceae) and *Ehrlichia* (Anaplasmataceae) are tick-borne pathogens that integrate the order Rickettsiales. They are obligate intracellular Gram-negative bacteria that multiply free in the cytosol (*Rickettsia*) or within vacuoles (*Ehrlichia*) of the host cells (Dumler et al., 2001). At least nine tick-borne *Rickettsia* species have been reported in Brazil, including *Rickettsia rickettsii* and *Rickettsia parkeri,* agents that cause spotted fever in humans, and the agents of unknown or uncertain pathogenicity, namely *Rickettsia amblyommatis, Rickettsia rhipicephali, Rickettsia monteiroi, Rickettsia bellii,* ‘*Candidatus* Rickettsia andeanae’, *Rickettsia* sp. strain Pampulha, and *Rickettsia* sp. strain Colombianensi’ (Parola et al., 2013; Luz et al., 2018; Nieri-Bastos et al., 2018).

At least three species of the genus *Ehrlichia* have been reported in Brazil. The most common ehrlichial agent in the country is *Ehrlichia canis,* the causative agent of canine monocytic ehrlichiosis, transmitted by the tropical lineage of the tick *Rhipicephalus sanguineus* sensu lato (Moraes-Filho et al., 2015). Recently, *Ehrlichia minasensis* was described infecting cattle and the tick *Rhipicephalus microplus* (Cruz et al., 2012; Aguiar et al., 2014; Cabezas-Cruz et al., 2016). Sacchi et al. (2012) reported *Ehrlichia chaffeensis* infecting marsh deer (*Blastocerus dichotomus*); however, its tick vector remains unknown in Brazil. In addition to these three *Ehrlichia* species, a number of novel ehrlichial genotypes have been recorded in Brazil, as for example, several phylogenetically closely related genotypes infecting jaguars (*Panthera onca*) (Widmer et al., 2011), peccaries (*Tayassu pecari*) (Soares et al., 2017), horses (Vieira et al., 2016), crab-eating fox (*Cerdocyon thous*) (Almeida et al., 2013), and sloth (*Bradypus tridactylus*) (Soares et al., 2017).

The Atlantic forest biome of Brazil is currently reduced to less than 6% of its original pre-Colombian extent, and currently exhibits high levels of forest fragmentation (da Silva and Casteleti, 2003). Several recent studies have provided evidence that degradation of the Atlantic forest has enhanced tick infestation on birds or small mammals, and in some cases, increased the exposure of domestic animals and humans to pathogenic or potentially pathogenic rickettsiae (Ogrzewalska et al., 2011, 2012; Dantas-Torres et al., 2012; Scinachi et al., 2017). In the case of small mammals, because of their limited dispersion and short lifespan, they can serve as good sentinels for the circulation of rickettsial agents in a particular area by natural environmental dispersion (Milagres et al., 2013). Besides, disorders in natural ecosystems can eventually bring humans into contact with wildlife-associated pathogens, resulting in the occurrence of emerging or re-emerging vector-borne diseases (Bradley and Altizer, 2007).

Most of the studies on tick-borne rickettsial organisms in Brazil have been done in the southern half of the country. In the northern half, studies have been concentrated in the Amazon biome, and a few ones in the semi-arid Caatinga biome. The scarcity of reports on tick-borne agents in the Atlantic forest remnants of northeastern Brazil prompted the current study, which evaluated infection by *Rickettsia* spp. and *Ehrlichia* spp. in small mammals and their ticks in two Atlantic forest conservation areas located in the state of Rio Grande do Norte, northeastern Brazil.

## 2 Materials and methods

### 2.1 Ethical statements

Procedures of this study have been previously approved by the “Instituto Chico Mendes” (ICMBio -SISBio permit 32104 -2), “Instituto de Desenvolvimento Sustentável e Meio Ambiente” of Rio Grande do Norte (IDEMA–RN), and by the Ethics Committee on Animal Use of the Institute of Biomedical Sciences, University of São Paulo, protocol number 204/2013.

### 2.2 Study area

The city of Natal, state of Rio Grande do Norte, northeastern Brazil, has 10 Environment Protection Zones (EPZ). This study was performed in two of these protected zones (EPZ-1: Parque da Cidade Dom Nivaldo Monte - 05°50′39.1″S 35°13′54.2″W; and EPZ-2: Parque Estadual das Dunas de Natal - 05°49′30.5″S 35°11′35.6″W), which have an Atlantic forest matrix as original biome; yet the ecosystems within consist of dune formations covered mostly with salt marsh vegetation peculiar to the Coastal Tablelands, Atlantic forest, and scattered patches of Caatinga vegetation. The climate is tropical humid with average annual temperature of 26 °C and annual rainfall of 2500 mm, with most intense rainy season between February and July (Freire, 1990; Ramalho and Pimenta, 2010).

### 2.3 Capture of small mammals and ticks

Two field campaigns were conducted to capture small mammals: one during the dry season (October 2012) and one at the beginning of the rainy season (February 2013). During two weeks (14 nights) per campaign, Sherman and Tomahawk-like traps, baited with a mixture of cornmeal, sardines and bananas were distributed alongside hiking passages within both parks, in sites where signs of animal activity was observed. A total of 40 traps per EPZ, distributed in four passages per park, were set at the first day. Traps were checked every morning, and baits were daily replaced.

Trapped animals were anesthetized with the association of xylazine (5 mg/kg) and ketamine (50 mg/kg), Afterwards, collection of blood was done by cardiac puncture, or from the tail or cephalic vein. Blood samples were allowed to clot at room temperature, and then centrifuged for separation of the serum, which was collected and kept frozen until serological analysis.

From 13 small mammals that were euthanized, lung and spleen fragments were collected for molecular analyses. The skins of the euthanized animals were deposited at the Museum of Natural History at the Pontifical Catholic University of Minas Gerais, Belo Horizonte City.

Every animal had the entire body examined for the presence of ticks, which were stored in absolute ethanol and brought to the laboratory. Morphological identification to species level of adult ticks of the genera *Amblyomma* and *Ixodes* followed Onofrio et al. (2006, 2009), whereas identification of *Amblyomma* nymphs followed Martins et al. (2010), and *Ornithodoros* larvae followed Kohls et al. (1969) and Barros-Battesti et al. (2013). Larvae of the genus *Amblyomma* were separated by morphotype and identified to species level by molecular analysis. For this purpose, *Amblyomma* larval DNA was tested by polymerase chain reaction (PCR) with primers 5′-CCG GTC TGA ACT CAG ATC AAG T-3′ and 5′-GCT CAA TGA TTT TTT AAA TTG CTG T-3′, which amplify a ≈460 bp of the tick mitochondrial 16S rRNA gene, as previously described (Mangold et al., 1998). PCR products were purified and sequenced in an automatic sequencer (model ABI 3500 Genetic Analyzer; Applied Biosystems/Thermo Fisher Scientific, Foster City, CA) according to the manufacturer's protocol. The generated sequences were submitted to BLAST analysis (www.ncbi.nlm.nih.gov/blast) to infer the closest similarities available in GenBank.

### 2.4 Serology for anti-Rickettsia spp. antibodies

The presence of anti-*Rickettsia* spp. IgG antibodies in the sera of the captured animals was assessed by immunofluorescence assay (IFA) using, simultaneously, crude antigens of six *Rickettsia* isolates from Brazil: *R. bellii* strain Mogi, *R. amblyommatis* strain Ac37, *R. rhipicephali* strain HJ5, *R. rickettsii* strain Taiaçu, *R. parkeri* strain At24, and *R. felis* strain Pedreira, as previously described (Labruna et al., 2007). Samples that reacted at the screening dilution (1:64) were then titrated using two-fold serial dilutions to determine the IgG endpoint titer. Slides were incubated with fluorescein isothiocyanate-labelled sheep anti-opossum IgG (CCZ, São Paulo, Brazil) for sera from marsupials, goat anti-rat IgG (Sigma, St Louis, MO, USA) for sera from Cricetidae rodents, and goat anti-guinea pig IgG (Sigma, St Louis, MO, USA) for sera from Caviomorpha rodents. In each slide, a serum previously shown to be non-reactive (negative control) and a known reactive serum (positive control) were tested at the 1:64 dilution. These sera derived from the studies of Horta et al. (2009) and Krawczak et al. (2016).

### 2.5 Molecular analyses of tick-borne bacteria

Ticks, and fragments of spleen and lung were submitted to DNA extraction by using the Wizard genomic DNA purification kit (Promega corporation, Madison, USA) following manufacturer's instructions. Adult ticks were tested individually; nymphs or larvae were processed in pools of three ticks from the same individual host. The concentration of extracted DNA was measured in a spectrophotometer UV (Bio Photometer plus, Eppendorf, Hamburg, Germany). Only samples with at least 20 ng/μl of DNA were subjected to PCR assays.

Tick DNA samples were tested by PCR using primers CS-78 (5′-GCA AGT ATC GGT GAG GAT GTA AT-3′) and CS-323 (5′-GCT TCC TTA AAA TTC AAT AAA TCA GGA T-3′), which amplify a 398-bp fragment of the citrate synthase gene (*gltA*) of all known *Rickettsia* species (Labruna et al., 2004). Samples yielding amplicon for this PCR assay were further tested by another PCR assay with primers Rr190.70F (5′-ATG GCG AAT ATT TCT CCA AAA-3′) and Rr190.701R (5′-GTT CCG TTA ATG GCA GCA TCT-3′), which amplify a 631-bp fragment of the 190-kDa outer membrane protein (*ompA*) of most of the spotted fever group *Rickettsia* species (Roux et al., 1996). In order to test the suitability of the DNA extraction protocol, tick samples with negative results for both rickettsial genes were further tested by the tick mitochondrial 16S rRNA gene PCR protocol described above.

Spleen and lung DNA samples of the small mammals were tested by PCR with primers EHR16SD-F (5′-GGT ACC YAC AGA AGA AGT CC-3′) and HE3 (5′-TGC ACT CAT CGT TTA CAG-3′), which amplify a 344-bp fragment of the 16S rRNA gene of *Anaplasmataceae* bacterial agents (Inokuma et al., 2000). Samples yielding amplicons by this PCR assay were further tested by a PCR assay with primers DSB-330 (5′-GAT GAT GTT TGA AGA TAT SAA ACA AAT-3′) and DSB-720 (5′-CTA TTT TAC TTC TTA AAG TTG ATA WAT C-3′), which amplify a 401-bp fragment of the *Ehrlichia* genus-specific disulfide bond formation protein gene (*dsb*) (Almeida et al., 2013). PCR products were purified, DNA-sequenced, and submitted to BLAST analysis as described above.

The *dsb* partial sequence of an erlichial agent detected in this study was aligned with corresponding sequences of different *Ehrlichia* species available in GenBank, using the T-COFFEE 8.93 program (McWilliam et al., 2013). A phylogenetic tree was inferred by Bayesian method with Mrbayes_3.2.5 software with 1,000,000 generations; the tree being sampled every 1000 generations, running 4 times beginning with random starting trees. The Jukes–Cantor model was used combined with the models of gamma distribution (G) (Huelsenbeck and Ronquist, 2001). The first 20% of the trees represented burning, and the remaining trees were used to calculate Bayesian posterior probability (BPP).

## 3 Results

A total of 39 small mammals were captured during the study, encompassing 33 marsupials (29 *Didelphis albiventris*, four *Monodelphis domestica*), three Cricetidae rodents (two *Necromys lasiurus*, one *Rattus rattus*), one Caviomorpha rodent (*Thrichomys apereoides*) and two armadillos (*Euphractus sexcinctus*). A total of 221 ticks were collected from 16 animals of only two mammal species, namely *D. albiventris* and *E. sexcinctus* (Table 1); no ticks were found on the remaining four mammal species. Three tick species, *Amblyomma auricularium, Ixodes loricatus,* and *Ornithodoros mimon* were collected from *D. albiventris,* whereas only *A. auricularium* was collected from *E. sexcinctus*. Taxonomic identification of *A. auricularium* larvae relied on morphological comparisons with lab-reared larvae from our laboratory (data not shown) and by molecular comparisons of three 16S rRNA gene partial sequences from three larval pools, which were identical to each other and 100% identical to a GenBank sequence of *A. auricularium* from northeastern Brazil (KR869154).

**Table 1:** Ticks collected from opossums (*Didelphis albiventris*) and armadillos (*Euphractus sexcinctus*) in two Environmental Protection Zones (EPZ-1 and EPZ-2) of Natal City, state of Rio Grande do Norte, northeastern Brazil.

Host species (No. captured)	Loca-lity	Ticks^a^									
		*Amblyomma auricularium*					*Ixodes loricatus*			*Ornithodoros mimon*	
		n	M	F	N	L	n	M	F	n	L
*D. albiventris* (19)	EPZ-1	5 (26)	1	1	5	16	0 (0)	0	0	3 (16)	19
*D. albiventris* (10)	EPZ-2	2 (20)	0	0	7	1	6 (60)	6	3	1 (10)	4
*E. sexcinctus* (2)	EPZ-1	2 (100)	15	6	107	30	0 (0)	0	0	0	0
Total (31)		9 (29)	16	7	119	47	6 (19)	6	3	4 (13)	23

a n: No. infested animals (%); M: No. males; F: No. females; N: No. nymphs; L: No. larvae.

Serum samples were collected from all captured animals, except for the two armadillos and one opossum. Overall, 6/28 (21%; confidence interval: 5.9%–36.1%) *D. albiventris* and the single *R. rattus* specimen reacted to at least one rickettsial antigen. Sera from four *M. domestica*, two *N. lasiurus*, and one *T. apereoides* did not react to any rickettsial antigen. Among the seven seroreactive animals, six (86%) reacted to *R. amblyommatis* (mean endpoint titers: 747; range: 128-2048), three (43%) to *R. bellii* (mean: 448; range: 64-1024), two (29%) to *R. rhipicephali* (mean: 160; range: 64-256) or *R. felis* (mean: 576; range: 128-1024), and one (14%) to *R. parkeri* (titer: 128); no serum reacted to *R. rickettsii.*

A total of 150 ticks (126 A*. auricularium,* nine *I. loricatus,* 15 *O. mimon*) was tested for rickettsial infection by PCR; 42 ticks (32 adults and 10 nymphs) were tested individually, and 108 ticks (nymphs and larvae) were tested in pools of three ticks each. While no rickettsia was detected in *I. loricatus* or *O. mimon*, most of the *A. auriculrium* ticks contained rickettsia (Table 2). Partial sequences of the *gltA* gene generated from 14 of these PCR-positive ticks (seven adults, three nymphal pools and one individual nymph, and two larval pools) were 100% (350/350 bp) identical to *R. amblyommatis* strain AaPE (KJ534310). All *gltA-*PCR positive tick samples yielded amplicon by the *ompA* PCR; from these, DNA sequences were generated from six adults, which were also 100% identical to *R. amblyommatis* strain AaPE (KJ534312). All rickettsia-negative tick samples yielded amplicons by the tick mitochondrial 16S rRNA gene PCR, validating the DNA extraction protocol.

**Table 2:** Results of molecular analysis for rickettsial infection in ticks collected from opossums (*Didelphis albiventris*) and armadillos (*Euphractus sexcinctus*) in two Environmental Protection Zones (EPZ-1 and EPZ-2) of Natal City, state of Rio Grande do Norte, northeastern Brazil.

Tick species	Tick stage	Loca-lity	Host	No. infected/No. ticks tested by PCR (%)	No. with DNA sequence^e^
*Amblyomma auricularium*	Adults	EPZ1	*E. sexcinctus*	21/21 (100)	6
	Nymphs			22/25 (88)^a^	3
	Larvae			3/3 (100)^b^	2
	Adults	EPZ1	*D. albiventris*	1/2 (50)	1
	Nymphs			2/3 (66)	1
	Larvae			0/3 (0)^c^	
	Nymphs	EPZ2	*D. albiventris*	3/7 (43)	1
*Ixodes loricatus*	Adults	EPZ2	*D. albiventris*	0/9 (0)	
*Ornithodoros mimon*	Larvae	EPZ1	*D. albiventris*	0/5 (0)^d^	

a tested in 25 pools of 3 ticks each (total: 75 nymphs), resulting in 22 PCR-positive pools.

b tested in 3 pools of 3 ticks each (total: 9 larvae), resulting in 3 PCR-positive pools.

c tested in 3 pools of 3 ticks each (total: 9 larvae), resulting in no PCR-positive pool.

d tested in 5 pools of 3 ticks each (total: 15 larvae), resulting in no PCR-positive pool.

e No. PCR-positive ticks or pools from which *gltA* partial sequence were generated, all 100% identical to *Rickettsia amblyommatis*.

**Fig. 1:**
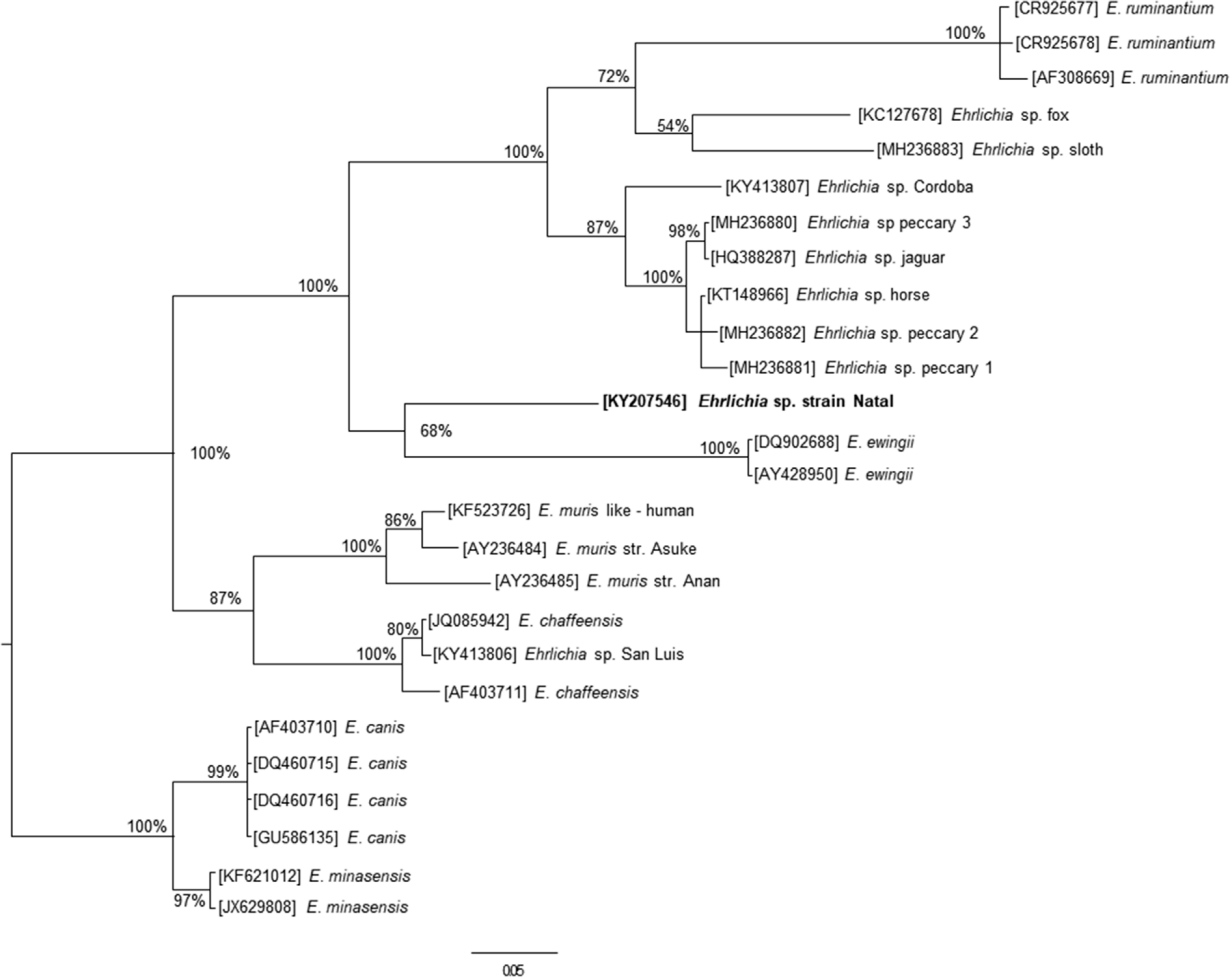
Bayesian analysis tree inferred from *dsb* gene partial sequences of *Ehrlichia* spp. Numbers at nodes are support values derived from posterior probability. The sequence obtained in this study (*Ehrlichia* sp. strain Natal) is in bold. Numbers in brackets are GenBank accession numbers. Scale bar: units of expected substitutions per site.

Lung and spleen samples were collected from 13 animals, namely two *N. lasiurus,* five *D. albiventris,* and three *M. domestica* from EPZ-1, and one *T. apereoides,* one *R. rattus,* and one *D. albiventris* from EPZ-2. Only the spleen from one *D. albiventris* from EPZ-1 yielded PCR amplicons for the ehrlichial genes 16S rRNA and *dsb*. DNA sequencing of the 16S rRNA amplicon generated a sequence that by BLAST analysis was closest (99% identity; 324/327 bp) to several uncultured *Ehrlichia* spp. from Oceania and Asia (MF069159, KR063138, FJ966352). The *dsb* sequence was closest (81%; 268/331 bp) to *E. chaffeensis* from the United States (JQ085942). The ehrlichial agent detected in opossum in the present study was named *Ehrlichia* sp. strain Natal. Phylogenetic analysis inferred from *dsb* partial sequences indicated that *Ehrlichia* sp. strain Natal was distinct from all known ehrlichial agents, since it formed an isolate clade, sister to *Ehrlichia ewingii* (Fig. 1). With 100% posterior probability, *Ehrlichia* sp. strain Natal and *E. ewingii* grouped in a clade composed by different haplotypes of *Ehrlichia ruminantium* and multiple haplotypes of unnamed ehrlichial agents that have been reported in Brazil and Argentina.

Novel DNA sequences generated in this study have been deposited in GenBank under accession numbers KY207547 and KY207546 for *Ehrlichia* sp. strain Natal 16S rRNA and *dsb*, respectively.

## 4 Discussion

The tick species *A. auricularium*, *I. loricatus* and *O. mimon*, have been previously reported in northeastern Brazil, where the same tick-host species associations found in the present study were recorded (Horta et al., 2011; Dantas-Torres et al., 2012; Saraiva et al., 2013; Labruna et al., 2014). In this study, most of the trapped small mammals were the white-eared opossum *D. albiventris*; very few rodents were trapped, in addition to the presence of an exotic species, the black rat *R. rattus*. These findings are compatible with Atlantic forest degradation, as previously reported (Bonvicino et al., 2002).

A high proportion of larvae, nymphs and adults of *A. auricularium* ticks collected from small mammals were found to harbor the spotted fever group agent *R. amblyommatis.* This finding is corroborated by a recent study that demonstrated 100% transovarial transmission and transstadial perpetuation of *R. amblyommatis* in *A. auricularium* ticks (Saraiva et al., 2013). Our serological analyses employing antigens of six *Rickettsia* species indicated highest seroprevalence and endpoint titers to *R. amblyommatis,* suggesting that at least part of the sampled small mammals have been infected by this agent, possibly via the parasitism by *A. auricularium* ticks. This statement is also corroborated by Saraiva et al. (2013), who demonstrated that this tick species was a competent vector of *R. amblyommatis*. Currently, *R. amblyommatis* is considered to be a potential human pathogen, based on serological evidence of human infection in the United States (Apperson et al., 2008; Vaughn et al., 2014). Noteworthy, one *A. auricularium* nymph was found attached to one of us (M.G.L.) during fieldwork (data not shown). Altogether, these results highlight the potential risk of human exposure to *R. amblyommatis* in the degraded Atlantic forest fragments of the present study.

A novel ehrlichial agent, *Ehrlichia* sp. strain Natal, was detected in an opossum *D. albiventris.* Phylogenetic analysis inferred from the highly polymorphic *dsb* gene of *Ehrlichia* spp. (Doyle et al., 2005) indicates that this novel agent is potentially a new species, yet to be formally described after in vitro isolation. All *Ehrlichia* species are known to be biologically transmitted by ixodid ticks (Dumler et al., 2001). Our acarological results incriminate two potential vectors of *Ehrlichia* sp. strain Natal, the ticks *I. loricatus* or/and *A. auricularium.* Further studies are needed to confirm this statement and the pathogenicity of *Ehrlichia* sp. strain Natal to animals and humans.

This work was performed in two environment protection zones of the Atlantic forest biome in northeastern Brazil, where human activities have almost completely destroyed or degraded the ecosystems during the last hundred years (da Silva and Casteleti, 2003). Even under such circumstances, the diversity of small mammals, their ticks, and potential tick-borne pathogens found in this study is noteworthy. Indeed, future studies should monitor the possible role of rickettsial and/or ehrlichial bacteria as agents of emerging diseases in these degraded areas of Atlantic forest, just as has occurred with other agents in degraded areas of this biome in southeastern Brazil (Ogrzewalska et al., 2012; Scinachi et al., 2017).

## Conflicts of interest

The authors declare no conflicts of interest.

## Appendix A Supplementary data

Data Profile Data Profile
